# BDNF and the maturation of posttranscriptional regulatory networks in human SH-SY5Y neuroblast differentiation

**DOI:** 10.3389/fncel.2014.00325

**Published:** 2014-10-15

**Authors:** Belinda J. Goldie, Michelle M. Barnett, Murray J. Cairns

**Affiliations:** ^1^The Centre for Translational Neuroscience and Mental Health, School of Biomedical Sciences and Pharmacy, University of Newcastle Callaghan, NSW, Australia; ^2^Schizophrenia Research Institute Sydney, NSW, Australia

**Keywords:** neuronal differentiation, MicroRNAs, gene expression profiling, cell culture techniques, SH-SY5Y cells

## Abstract

The SH-SY5Y culture system is a convenient neuronal model with the potential to elaborate human/primate-specific transcription networks and pathways related to human cognitive disorders. While this system allows for the exploration of specialized features in the human genome, there is still significant debate about how this model should be implemented, and its appropriateness for answering complex functional questions related to human neural architecture. In view of these questions we sought to characterize the posttranscriptional regulatory structure of the two-stage ATRA differentiation, BDNF maturation protocol proposed by Encinas et al. (2000) using integrative whole-genome gene and microRNA (miRNA) expression analysis. We report that ATRA-BDNF induced significant increases in expression of key synaptic genes, brain-specific miRNA and miRNA biogenesis machinery, and in AChE activity, compared with ATRA alone. Functional annotation clustering associated BDNF more significantly with neuronal terms, and with synaptic terms not found in ATRA-only clusters. While our results support use of SH-SY5Y as a neuronal model, we advocate considered selection of the differentiation agent/s relative to the system being modeled.

## Introduction

The nervous system, and in particular the brain, arguably represents the most complex area of human biology. Many different cell types are present, interacting in multiplexed combinations, and every circuit is uniquely wired by individual patterns of experience. This complexity continues down to the subcellular level with intricate transportation systems and biomolecular partitioning. Temporo-spatial specificity of mRNA translation in particular is critical for localized protein synthesis as it supports the synaptic remodeling required for synaptic plasticity.

Recent studies have suggested that small, non-coding RNA species known as microRNA (miRNA) play a role in supporting temporo-spatial traffic of neuronal mRNA (Goldie and Cairns, 2012). Many miRNA demonstrate brain-specific (Smirnova et al., 2005; Krichevsky et al., 2006) and region-specific (Hollins et al., 2014) expression and have been shown to regulate key aspects of brain development and neuronal morphology (Giraldez et al., 2005), including patterning and arealisation, dendritic branching (Xu et al., 2008) and spine volume (Schratt et al., 2006). The presence of miRNA in exosomes suggests they may also play a role in activity-driven communication (Fauré et al., 2006).

A key limitation in studies of the nervous system is that ethical considerations and availability of tissue generally restrict these investigations to animal models, relying on *in-vitro* or *ex-vivo* preparations, typically of mouse or rat, to extrapolate human function. Although the composition of the post-synaptic density is well conserved among mammals (Bayés et al., 2010), the dynamic component of plasticity provided by mRNA and miRNA is quite divergent. Human transcription networks have evolved a complexity that drives species-specific gene expression in the pre-frontal (PFC) and frontal cortices (Konopka et al., 2012). Among transcripts that are subject to human-specific developmental remodeling, miRNA in the PFC exhibit the greatest and most rapid divergence from other primates with an average evolutionary length 24 times that of other transcript types (Somel et al., 2011). These findings suggest that animal models, while informative, may not provide full understanding of the molecular mechanisms underlying neuronal function in higher primates and humans.

## Neuronal differentiation of neuroblastomas

To overcome this limitation, several human immortalized cell lines are available which for some purposes can model human neuron behavior. Among these, neuroblastoma cell lines are highly accessible and can be matured into terminally differentiated, neuron-like cells using agents such as retinoic acid (RA), enabling clearer determination of molecular and morphological changes induced by differentiation. Treatment with RA is associated with inhibition of proliferation, extension of processes commonly termed neurites (Stio et al., 2001), increased acetylcholinesterase (AChE) activity (Sidell et al., 1984), and enhanced production of synaptic vesicles (Sarkanen et al., 2007); features consistent with neuronal maturation.

In the SH-SY5Y cell line, a sympathetic line containing noradrenaline and neuropeptide Y in dense-core vesicles (Goodall et al., 1997; Ou et al., 1998), three stereoisomers of RA, *all-trans* (ATRA), *9-cis* and *13-cis*, are all observed to induce this phenotype, however differential activity at the receptor level appears to impact the differentiation process. ATRA acts at the native retinoic acid receptor (RAR), which binds to retinoic acid response elements (RAREs) in the DNA to alter transcription of RA-activated genes, while the *9-cis* isomer acts in a similar manner on the retinoid X receptor (RXR). This difference could explain the observation that although *9-cis* RA induces stronger morphological and transcriptional responses these changes are reversed after washout, while the response to ATRA is permanent (Redfern et al., 1994). This finding suggests that ATRA may be a more suitable agent for studying the effects of commitment to a neuronal lineage.

## Neuronal maturation with BDNF

In the developing brain, the final connectivity of a neuron is determined largely by signals received from the neurotrophin family of proteins, which are expressed in a laminar-specific pattern and influence the growth and complexity of the dendritic tree (McAllister et al., 1995). Of this family, brain-derived neurotrophic factor (BDNF) has a profound effect on pyramidal neurons in cortical layers 4 and 5, in addition to which it has demonstrated the capacity to target mRNAs to the synapse (Tongiorgi et al., 1997; Righi et al., 2000) and stimulate their local translation (Miyata et al., 2005). In particular, polysome profiling of active translation conducted by Schratt and colleagues demonstrated that BDNF is required to stimulate local translation of key synaptic components such as CamKII, NMDA receptors NR1, and NR3, PSD93 and LIMK-1 (Schratt et al., 2004). These findings suggest the employment of BDNF for *in vitro* neuronal maturation may produce cells more closely matching the phenotype and, importantly, gene expression profile of neurons *in vivo*.

BDNF is active at the *trkB* receptor (gene name NTRK2), which is absent from naïve SH-SY5Y cells; its expression can be induced by differentiation with ATRA (Kaplan et al., 1993), and some studies have employed a 5–6 day protocol of concurrent ATRA and BDNF treatments. However, in an arguably seminal paper published in 2000, Encinas and colleagues demonstrated that *trkB* does not reach peak expression in this cell line until 5 days' exposure to ATRA (Encinas et al., 2000); thus these short-duration, dual-agent protocols may bear limited similarity to mature neurons: a model of sequential treatment with ATRA for 5 days followed by BDNF, that yields neurotrophin-dependent mature cells, may be more physiological.

## Rationale and methods

We performed a comprehensive review of the literature to understand the use of differentiation protocols for the maturation of SH-SY5Y cells. Specifically, for *in vitro* research work attempting to emulate mature neuronal behavior, we wanted to know to what extent the sequential treatment with ATRA followed by BDNF was being utilized compared with other differentiation protocols or undifferentiated cells. A search of the PubMed literature for “SH-SY5Y” identified 3419 papers published in English between the publication of the Encinas protocol (2000) and the time of writing (April 2014), from which 410 papers studying the neuroblastoma disease itself were excluded. This list was narrowed to 2307 papers by searching for words associated with differentiation, neurodegeneration and mental health conditions (see Supplementary Figure S1 for full description of review inclusion/exclusion criteria). Surprisingly, 1914 of 2307 papers (83%) did not utilize a differentiation protocol at all, nor was an explanation for using undifferentiated cells given.

Among 393 studies employing a differentiation protocol, the most common differentiation agent was ATRA only (283 of 393 papers, 72%, or 12% of all studies); approximately 16% (65 papers, 3% of all studies) utilized other differentiating agents such as the phorbolester 12-O-tetradecanoylphorbol-13-acetate (TPA), which has been shown to generate an adrenergic phenotype (Påhlman et al., 1983). Moreover, a remarkably small proportion (45 papers, 11 or 2% of all studies) utilized Encinas' sequential protocol of ATRA differentiation followed by BDNF, despite its compelling evidence: The switch from ATRA to BDNF on day 5 coincides with peak expression of its receptor *trkB*, however the biochemical impact of this transition has not previously been reported, nor has the gene expression profile been extensively characterized. Moreover, the importance of post-transcriptional regulation of gene expression by miRNA in the control of neuronal differentiation, development, connectivity, and synaptic function is now well established, as reviewed in Goldie and Cairns (2012); a thorough investigation of this two-stage neuronal model in this light is therefore timely.

We undertook comparative genome-wide gene and miRNA expression analyses of naïve, 5-day ATRA-treated and 5-day ATRA- + 7-day BDNF-treated SH-SY5Y using Affymetrix Exon v1.0 and miRNA v2.0 microarrays. Data were analyzed with Genespring Gx 12 software (Agilent) to identify differentially expressed transcripts with Benjamini-Hochberg correction for multiple testing and a corrected *p*-value cut-off of 0.05. Microarray results were confirmed by examination of a selection of significantly altered transcripts by qPCR with random-primed cDNA for gene expression and specific mature miRNA primers for miRNA expression as described previously (Santarelli et al., 2011). Functional analysis of gene expression changes was conducted using the Functional Annotation Clustering (FAC) tool of the DAVID bioinformatics suite (Huang et al., 2008) on lists of genes having significantly different expression (as defined above) at each timepoint. Integrated analysis of altered miRNA expression was conducted using QIAGEN's Ingenuity Pathways Analysis software (IPA, QIAGEN Redwood City, www.qiagen.com/ingenuity). A target analysis was performed on significantly up- and down-regulated miRNA, and the list of targets refined by expression pairing with the list of altered genes. Core analysis was carried out on negatively correlated pairings to investigate significantly altered pathways and functional networks.

To report on biochemical neuronal maturity, we measured the acetylcholinesterase (AChE) activity of samples collected at various timepoints during differentiation and maturation using the Amplex Red AChE assay kit according to the manufacturer's instructions (Invitrogen).

## Sequential differentiation with ATRA then BDNF yields a more neuronal cell population

### Morphology

**Figure 1:**
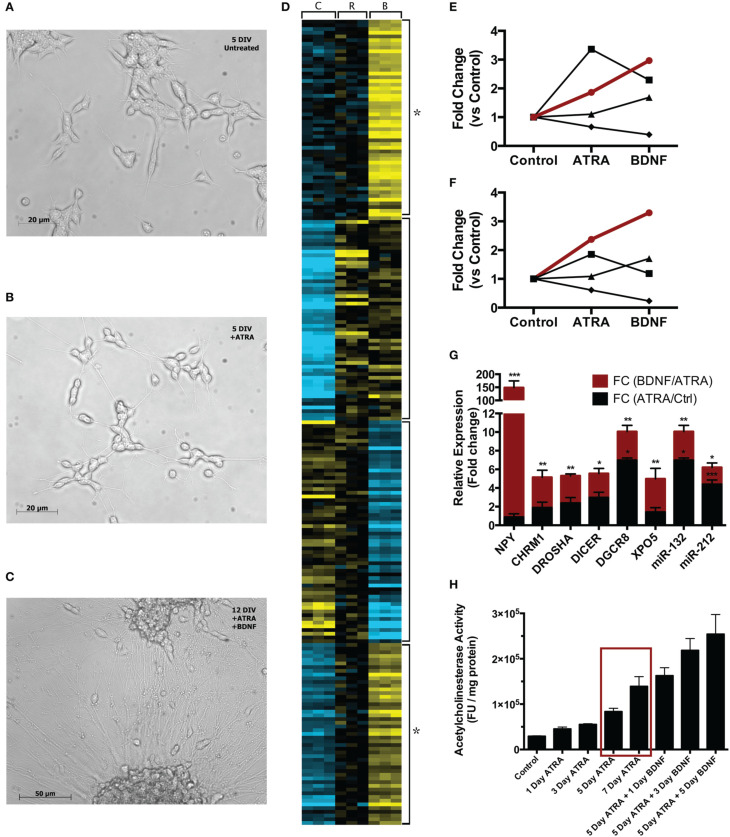
**Comparison of single-stage (ATRA) and two-stage (ATRA + BDNF) differentiation of SH-SY5Y**. Compared with **(A)** 5 DIV untreated cultures, cells **(B)** treated with ATRA for 5 days demonstrate reduced proliferation and take on a more polar appearance, with neurites being extended and networks beginning to develop. **(C)** After further maturation with BDNF, cell bodies migrate into clusters and the neurite networks become increasingly complex. **(D)** Heat map (*k*-means, 4 clusters) clustering genes and miRNA significantly differentially expressed (blue = low expression, yellow = high) during sequential differentiation. C = control, R = ATRA, B = BDNF. Four clusters reveal key expression patterns, in particular the strength of BDNF induction (star). These are summarized as trend lines showing average expression of constituent **(E)** genes and (**F**) miRNA; the groups of transcripts demonstrating two-stage induction (red lines) clustered significantly for neuronal functional terms (see Table 1A) and contained many key neuronal peptides, receptors and miRNA (see Table 1B). Symbols delineate differing two-stage expression patterns. **(G)** A selection of these transcripts examined by qPCR confirmed that maturation with BDNF drove increased expression (red bars) compared with ATRA differentiation alone (black bars). ^*^p < 0.05; ^**^p < 0.01; ^***^p < 0.001. **(H)** AChE activity was measured after 1, 3, 5, and 7 days' ATRA treatment, or 5 days' ATRA treatment followed by 1, 3 or 5 days' treatment with BDNF. Increases in AChE activity were most strongly elicited by BDNF maturation; notably, 5 day ATRA + 1 day BDNF evoked more response than an extended 7 day ATRA treatment (red box). Treatments were 10 μM ATRA and 50 ng/ml BDNF throughout.

Neuroblast cultures were imaged at each stage of treatment at 10X magnification with an Axiovert inverted microscope (Zeiss) (Figure 1). In line with the results of Encinas and colleagues, cells visually appeared more neural across the two-stage differentiation process. Compared with naïve cells (Figure 1A), cells treated with ATRA for 5 days appeared to have reduced proliferation, took on a more polar morphology and began to extend longer, more robust neurites (Figure 1B). During 7 days subsequent BDNF exposure, cell bodies began to migrate into clusters, some a large as ~110 μm diameter, while neurites increased in number, size and complexity (Figure 1C).

### Gene expression

Differentiation with ATRA significantly altered the expression of 46 genes; remarkably, 38 of these were up regulated, characterizing neuronal differentiation as the “switching on” of a genetic programme. BDNF maturation had a much bigger effect on gene expression, significantly altering 265 genes (189 up, 76 down) compared with controls and, importantly, 387 genes (123 up, 264 down) compared with ATRA differentiated samples. As shown in Figure 1D, the genes most strongly induced by ATRA were down regulated by BDNF maturation, while a group showing only mild ATRA induction were further strengthened by BDNF. These included many neuronal genes and components of the miRNA biogenesis machinery (Table 1A), a panel of which were validated by qPCR (Figure 1F). Strikingly, neuropeptide Y (NPY), which has been reported as non-responsive to ATRA (Påhlman et al., 1995), demonstrated 146-fold increase in expression in response to sequential ATRA + BDNF.

**Table 1A:** **Neuronal genes and miRNA demonstrating increased expression after BDNF maturation of ATRA-differentiated SH-SY5Y**.

**Gene symbol**	**Gene name**	**Fold-change (BDNF/ATRA)**
NPY^a^,^c^	Neuropeptide Y	146
RELN^a^,^b^,^d^,^e^	Reelin	9.01
NTRK2^b^,^d^	Neurotrophic tyrosine kinase, receptor, type 2	2.0
GRIK3^a^,^d^,^f^	Glutamate receptor, ionotropic, kainate 3	3.3
GRIN2B^a^,^b^,^c^,^d^	Glutamate receptor, NMDA, subunit 2B	3.28
CHRM1^a^,^b^	Cholinergic receptor, muscarinic 1	3.23
HTR3C^a^	Serotonin receptor 3C	3.28
HTR5A^a^,^d^,^e^	Serotonin receptor 5A	3.3
DRD2^a^,^c^,^f^	Dopamine receptor D2	3.3
HCRTR1^d^,^e^	Hypocretin (orexin) receptor 1	3.1
CACNA1C^a^,^b^,^d^,^e^	Calcium channel, voltage-dependent, 1C subunit	3.3
NRG1^a^,^b^,^d^	Neuregulin 1	3.25
DICER1	Dicer 1, ribonuclease type III	2.56
DROSHA^a^	Drosha, ribonuclease type III	2.94
DGCR8^a^	DiGeorge syndrome critical region gene 8	3.04
XPO5	Exportin 5	3.48
MIR132	Hsa-miR-132	3.04
MIR212	Hsa-miR-212	1.78

*Disease associations:*

a *schizophrenia*.

b *Alzheimer*.

c *Huntington's*.

d *bipolar/mood disorders*.

e *autism spectrum disorders*

f *Parkinson's*.

Genes “activated” by each of the ATRA differentiation and BDNF maturation processes were functionally analyzed using the DAVID FAC tool. In ATRA-treated samples the only cluster terms to remain significant after *p*-value correction related to retinoid metabolism. This is perhaps not surprising given the subsequent down regulation of genes induced by ATRA treatment. In contrast, when compared with both control and ATRA-treated cohorts, BDNF-matured samples were strikingly enriched for neuronally relevant clusters including “neuron projection” (*ES* = 4.83, corrected *p* = 0.00027) and “synaptic transmission” (*ES* = 2.33, corrected *p* = 0.03). Although some of these terms were present in the ATRA results, they were not significant (Table 1B).

**Table 1B:** **Comparison of significance of neuronal terms derived from functional clustering of genes altered by sequential differentiation and maturation of SH-SY5Y**.

**Term**	**Corrected *p*-value**		
	**ATRA vs CTRL**.	**BDNF vs CTRL**.	**BDNF vs ATRA**.
Neuron development	0.238	0.037	5.19E-03
Neuron differentiation	0.397	3.05E-03	6.24E-03
Axon	0.557	3.04E-05	0.019
Axonogenesis	0.750	0.046	2.40 E-03
Cell morphogenesis involved in neuron differentiation	0.763	0.066	2.31 E-03
Neuron projection	0.769	2.52E-05	2.65E-04
Neuron projection development	0.812	0.065	3.94E-03
Synaptic transmission	n/a	1.25E-04	0.031
Synapse	n/a	7.92E-05	0.079

### miRNA expression

In response to ATRA differentiation, 36 miRNA demonstrated significantly different expression. Unlike gene expression there was no clear directional bias with 20 up and 16 down regulated. Also in contrast to the gene expression results, the miRNA most strongly induced by ATRA were further strengthened by BDNF maturation (Figure 1E). BDNF induced differential expression of 70 miRNA compared to undifferentiated cells (42 down, 28 up). Notably, differentiation dramatically increased the expression of miR-132, a key regulator of dendritic growth and arborisation, as well as known brain-specific miRs –210 and –212, the latter of which inhabits a locus with miR-132. Significant increase in expression of miRs –132 and –212 by BDNF maturation was confirmed by qPCR (Figure 1F). We also observed ATRA-mediated decrease in miR-17 family expression, consistent with previous observations (Beveridge et al., 2009); all members of this family were further down-regulated by BDNF maturation.

### Functional integration

The importance of BDNF in shaping the regulatory environment of matured cells was investigated using IPA software. Genes showing two-stage activation (173 genes, Figure 1E, red line) were paired with a target analysis of 44 negatively correlated miRNA (10 miRNA with 42 target genes). Core analysis of these molecules showed a strong enhancement of neuronal functionality, with top functional terms “neurotransmission” (*p* = 1.97e-07, APP, GRIK2, KCNMB4, PCDHB10, PCDHB14, PCDHB5, PSEN1), “neurological disease” (*p* = 7.41e-07, APP, BCL2, CHGB, DPYSL3, ESRRG, GABRA3, GRIK2, LGR5, NTRK2, PLK2, PSEN1, SCN3A, VCAN), “synaptic transmission of cells” (*p* = 1.52e-06, APP, GRIK2, PCDHB10, PCDHB14, PCDHB5, PSEN1), and “synaptogenesis” (*p* = 1.93e-06, APP, PCDHB10, PCDHB14, PCDHB5). Full results are presented in Supplementary Table S1. Visual representation of this analysis revealed that many of these genes encode proteins that reside in the synaptic membrane (Supplementary Figure S2). Moreover, four families of miRNA (miR-18a,b, miR-17,20a,b, miR-130, and miR-1275) were found to be key hubs providing regulatory support to this functionality.

### Acetylcholinesterase activity

Only modest changes in AChE activity were observed during ATRA-induced differentiation, however maturation with BDNF caused sharp increases in activity (Figure 1G). Strikingly, AChE activity in cells treated with ATRA for 5 days followed by 1 day of BDNF exposure was higher than in those treated for 7 days with ATRA alone, highlighting the importance of the neurotrophin in development of the neuronal phenotype. Moreover, the magnitude of the response increased with ATRA concentration used to induce differentiation (Supplementary Figure S3), possibly due to the expression of more BDNF receptors at higher ATRA concentrations.

## Discussion

Human neuroblast cultures, particularly the SH-SY5Y line, are used extensively to model the basic biology of neurons. They may also have application in high-throughput screening assays of neuroactive compounds. The literature is controversial regarding the need to differentiate SH-SY5Y, as exemplified by an exchange between Luchtman and Song (2010) regarding the latter's conclusions with respect to the applicability of SH-SY5Y differentiation in studying neurotoxicity and neuroprotection in Parkinson's disease (Cheung et al., 2009). What is clear from this exchange is the need to carefully consider experimental methodology and applicability of the cell model used within the context of the research question.

Our results presented here suggest that the sequential ATRA differentiation-BDNF maturation program yields a cell population with significantly more characteristics of mature neurons compared with ATRA treatment alone. We observed obvious morphological similarities with neurons, including intricate and complex neurite structure and evidence of migration with cell bodies organizing into clusters. The cells also displayed substantial enhancement of neuron-associated gene expression, in particular induction of NPY, NTRK2, and CHRM2. There was also evidence of increased posttranscriptional regulation, with elevated expression of miRNA biogenesis machinery including, DROSHA, DGCR8, DICER, and XPO5. This capacity was realized through significantly elevated expression of many miRNA, including key neuronal miRs –132 and –212, supporting the critical roles these molecules play in developing and mature neurons. These individual findings were corroborated at the systems level by the abundance of terms related to neuronal operation and synapse formation derived from the integrated functional analysis. Moreover we have recently shown that miRNA expression and distribution in these cells is rapidly altered in response to potassium-induced depolarisation (Goldie et al., 2014). A substantial component of this depolarisation-associated change in miRNA was found to be mediated by release of miRNA-enriched exosomes.

These higher orders of neural differentiation and function are dependent on neurotrophin signaling. The importance of BDNF and the expression of the *trk* receptors in shaping the neuronal specialization have been demonstrated from development through maturity and plasticity. In particular, during cortical development this neurotrophin exerts profound effects on layer 4 and 5 neurons, where it significantly increases the number and complexity of dendritic branches (McAllister et al., 1995), axonal length (Labelle and Leclerc, 2000), as well as the number of spines (Bamji et al., 2006) and therefore potential synaptic connectivity. BDNF maturation of SH-SY5Y clearly reproduced this effect in terms of neurite morphology; however it is generally considered that these cells do not form synapses. Studies in neurons have shown that the trkB receptor is required for synapse formation, and ablation of trkB resulted in deficits of synapse formation (Luikart et al., 2005). Further, the interaction between BDNF and the trkB receptor plays an important role in neuronal development (Cheng et al., 2011; Dong et al., 2012), and is important for synaptic plasticity (Kang et al., 1996).

Our systematic review revealed only 2% (45 of 2307) of papers used sequentially differentiated cells, and only 35 of these studies allowed sufficient exposure to ATRA (5 days) to allow for maximal expression of trkB. Without the interaction between BDNF and trkB, which is optimally induced by exposing the cells to ATRA for 5 days, synapse formation is unlikely and has not been reported in conventional culture. However, by pre-treating these cells with ATRA, Agholme and colleagues were able to detect synaptic structures and vesicular transport in a 3D gel matrix culture. Synapses were found in samples matured in BDNF alone, however were more numerous when matured with a cocktail containing BDNF, neuregulin β 1, nerve growth factor and vitamin D_3_ (Agholme et al., 2010). Although more work needs to be done to fully characterize these structures, this finding indicates that with more development this culture technique may provide a system for unraveling some of the complexities of the human neuron maturation and connectivity.

## Conclusions

SH-SY5Y is a flexible culturing system that can be differentiated into several mature neuronal phenotypes, depending on the differentiation agent selected. In our investigation of gene expression and the posttranscriptional regulatory environment of these cells, we found that differentiation combined with BDNF maturation is optimal for generating a phenotype approaching mature neurons. By contrast, the changes induced by ATRA alone appeared to produce an intermediate phenotype between immature neuroblasts and mature neurons, and may be a suitable analog for the study of developing neurons. This system provides a robust *in vitro* alternative to animal models for investigating some aspects of neuronal function with the advantage of the human genetic context for revealing species-specific higher order complexity.

### Supplementary material

The Supplementary Material for this article can be found online at: http://www.frontiersin.org/Behavioral_Neuroscience/10.3389/fnbeh.2012.00089/abstract

### Conflict of interest statement

The authors declare that the research was conducted in the absence of any commercial or financial relationships that could be construed as a potential conflict of interest.
