# Supplementary material for: BDNF and the maturation of posttranscriptional regulatory networks in human SH-SY5Y neuroblast differentiation
Source: Front Cell Neurosci. 2014 Oct 15;8:325. doi: 10.3389/fncel.2014.00325 (PMC4197648; doi:10.3389/fncel.2014.00325)
Supplement: Supplementary file 1 [file Table1.DOCX]

**Supplementary Figure S2. Down-regulation of key miRNA accompanies up-regulation of genes integral to neuronal phenotype.** IPA target analysis of miRNA incrementally down regulated during two-stage differentiation was integrated with up-regulated mRNA and overlaid with cellular functions statistically over-represented among targeted genes. This sub-cellular layout demonstrates the functional importance of four families of miRNA in regulating many mRNA central to neuron development, with an emphasis on the composition of the synaptic membrane.

**Supplementary Figure S3. Enhanced neuronal maturity is primed by ATRA concentration.** Cells were differentiated with titrated concentrations of ATRA followed by maturation with the same concentration of BDNF. Neuronal maturity was assayed by AChE activity at various timepoints during BDNF maturation and the values averaged. A very strong correlation was observed between concentration of ATRA used for differentiation and the level of BDNF induced activity, suggesting that the production of more BDNF receptors enhances neuronal maturity.
