# Supplementary figures and images for: BDNF and the maturation of posttranscriptional regulatory networks in human SH-SY5Y neuroblast differentiation
Source: Front Cell Neurosci. 2014 Oct 15;8:325. doi: 10.3389/fncel.2014.00325 (PMC4197648; doi:10.3389/fncel.2014.00325)

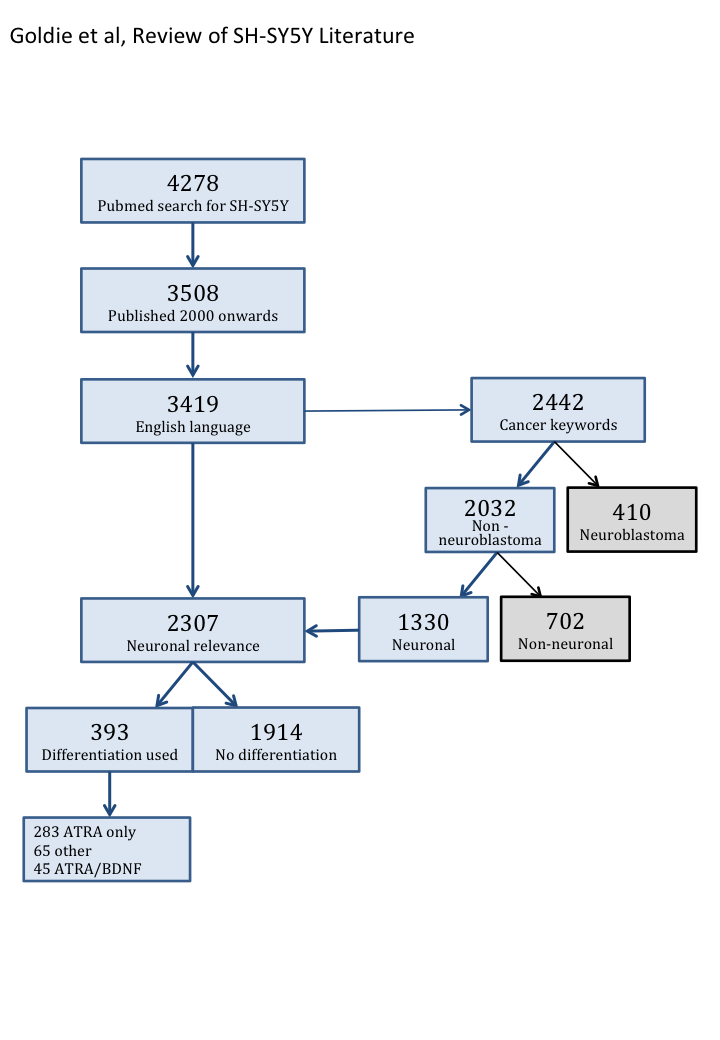

Supplement: Supplementary file 3 [file Image1.TIF]

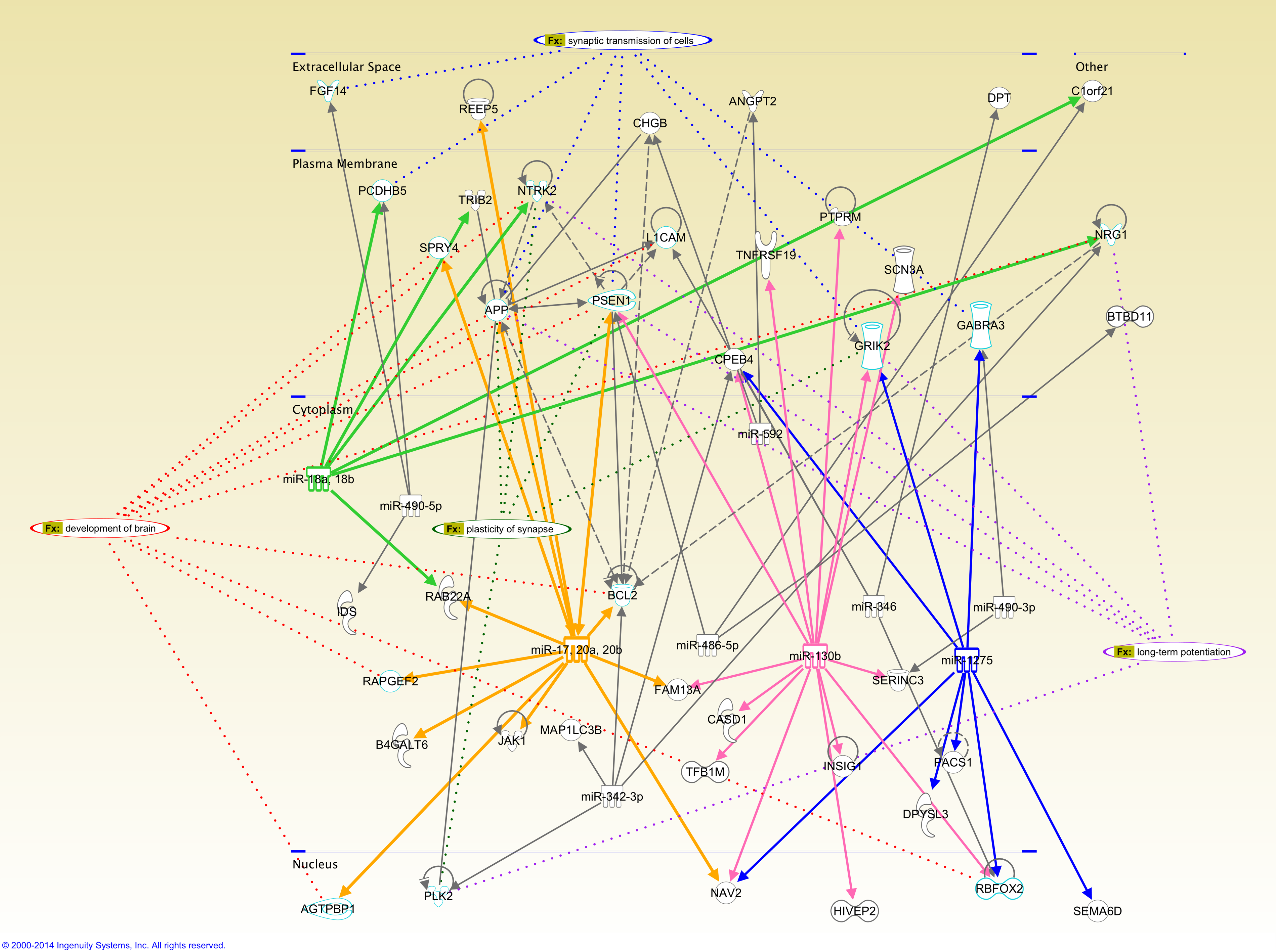

Supplement: Supplementary file 4 [file Image2.TIFF]

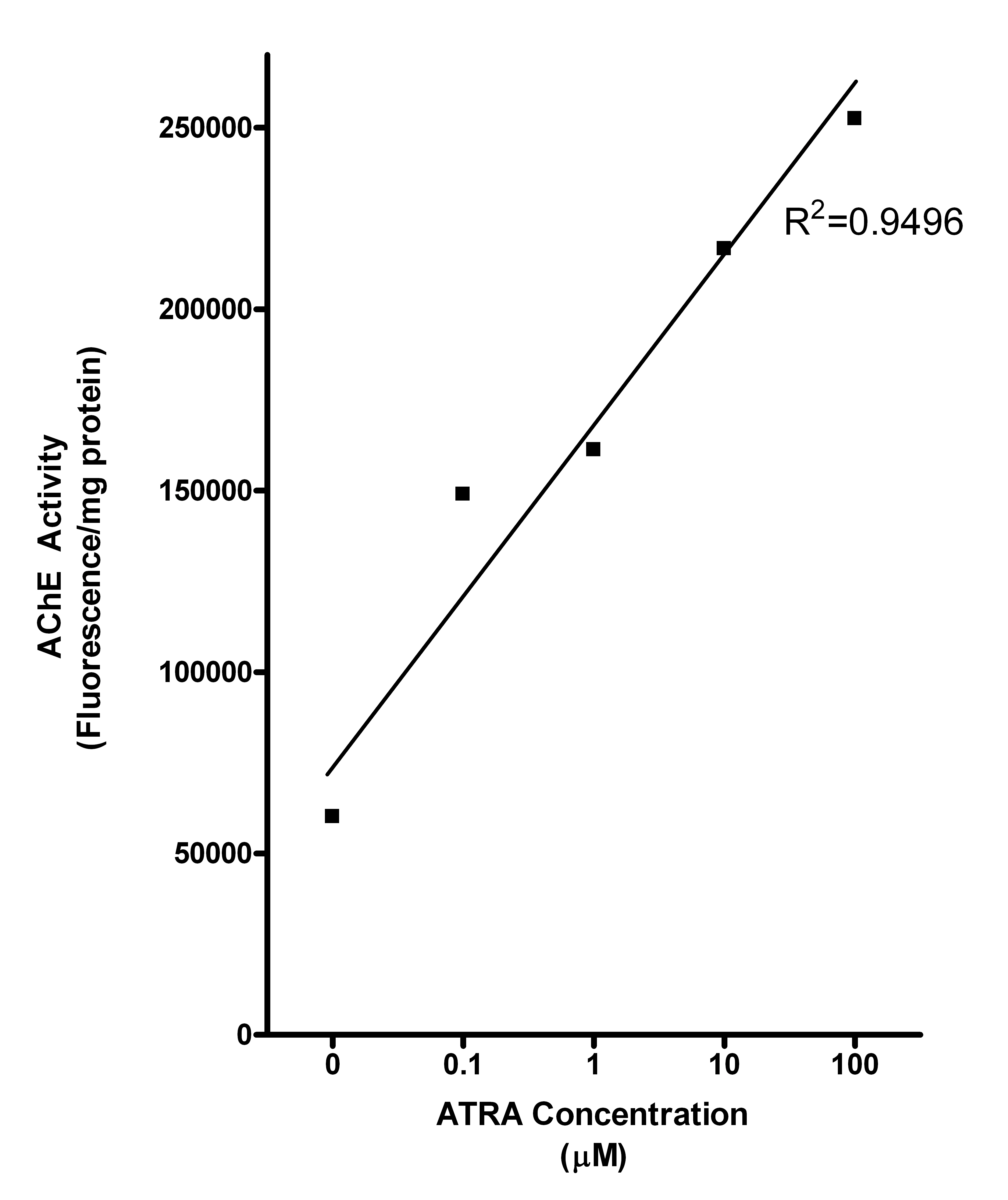

Supplement: Supplementary file 5 [file Image3.JPEG]
